# Antibody induced seizure susceptibility and impaired cognitive performance in a passive transfer rat model of autoimmune encephalitis

**DOI:** 10.3389/fimmu.2023.1268986

**Published:** 2023-11-15

**Authors:** Şura Akat Pişkin, Hande Yüceer Korkmaz, Canan Aysel Ulusoy, Elif Şanlı, Cem Ismail Küçükali, Filiz Onat, Erdem Tüzün, Nihan Çarçak

**Affiliations:** ^1^ Department of Pharmacology, Faculty of Pharmacy, Institute of Health Sciences, Istanbul University, Istanbul, Türkiye; ^2^ Department of Neuroscience, Aziz Sancar Institute of Experimental Medicine, Institute of Health Sciences, Istanbul University, Istanbul, Türkiye; ^3^ Department of Neuroscience, Aziz Sancar Institute of Experimental Medicine, Istabul University, Istanbul, Türkiye; ^4^ Department of Medical Pharmacology, Acibadem Mehmet Ali Aydinlar University Faculty of Medicine, Istanbul, Türkiye; ^5^ Deparment of Neuroscience, Acibadem Mehmet Ali Aydinlar University Health Sciences Institute, Istanbul, Türkiye; ^6^ Department of Pharmacology, Faculty of Pharmacy, Istanbul University, Istanbul, Türkiye

**Keywords:** autoimmune encephalitis, NMDAR antibody, Epilepsy, LGI1, Wistar, PTZ, animal model

## Abstract

**Objective:**

Autoimmune encephalitis (AE) is a distinct neuro-immunological disorder associated with the production of autoantibodies against neuronal proteins responsible for pharmacoresistant seizures, cognitive decline and behavioral problems. To establish the causal link between leucine-rich glioma inactivated 1 (LGI1) antibody and seizures, we developed an *in-vivo* antibody-mediated AE rat model in which serum antibodies (IgG) obtained from blood samples of leucine-rich glioma inactivated 1 (LGI1) protein antibody (IgG) positive encephalitis patients were passively transferred into non-epileptic Wistar rats. Serum IgG of N-methyl-d-aspartate receptor (NMDAR) antibody positive patients were used as positive control since the pathogenicity of this antibody has been previously shown in animal models.

**Methods:**

Total IgG obtained from the pooled sera of NMDAR and LGI1-IgG positive patients with epileptic seizures and healthy subjects was applied chronically every other day for 11 days into the cerebral lateral ventricle. Spontaneous seizure development was followed by electroencephalography. Behavioral tests for memory and locomotor activity were applied before and after the antibody infusions. Then, pentylenetetrazol (PTZ) was administered intraperitoneally to evaluate seizure susceptibility. Immunohistochemistry processed for assessment of hippocampal astrocyte proliferation and expression intensity of target NMDAR and LGI1 antigens.

**Results:**

No spontaneous activity was observed during the antibody infusions. PTZ-induced seizure stage was significantly higher in the NMDAR-IgG and LGI1-IgG groups compared to control. Besides, memory deficits were observed in the NMDAR and LGI1-IgG groups. We observed enhanced astrocyte proliferation in NMDAR- and LGI1-IgG groups and reduced hippocampal NMDAR expression in NMDAR-IgG group.

**Significance:**

These findings suggest that neuronal surface auto-antibody administration induces seizure susceptibility and disturbed cognitive performance in the passive transfer rat model of LGI1 AE, which could be a potential *in-vivo* model for understanding immune-mediated mechanisms underlying epileptogenesis and highlight the potential targets for immune-mediated seizures in AE patients.

## Introduction

Autoimmune Encephalitis (AE) is recently identified immune-mediated disorder apparently accounting for 10-15% of all encephalitis cases (1). According to recent hypotheses, seizures occurring in the context of AEs could be provoked by the direct effect of auto-antibodies (auto-Abs) on neuronal surface antigens (e.g., ion channels, receptor proteins) involved in synaptic transmission (2, 3). Auto-Abs against N-methyl-d-aspartate receptor (NMDAR) and leucine-rich glioma inactivated 1 (LGI1) proteins expressed on the neuronal surface resulting in hyperexcitability and impairment of synaptic function are considered to be pathogenic in patients with AE that is characterized by a wide range of neurological and psychiatric clinical features including cognitive impairment, behavioral changes, movement disorders and epileptic seizures (4). Although immunotherapy improves clinical outcomes, some individuals may experience deficiencies that result in lifelong disability, such as pharmacoresistant epilepsy and persistent cognitive impairment (3). The detection of particular auto-Abs found in serum and/or cerebrospinal fluid (CSF) of patients in conjunction with an appropriate clinical presentation is required for the diagnosis of AE. However, in a considerable number of patients no auto-Abs expressed and presumably not all antibodies are known (4). Experimental studies conducted with antibodies from patients with NMDAR encephalitis also suggest that antibodies against neuronal cell-surface may contribute to epileptogenesis (5–8). However, it remains unknown in what extend the LGI1-directed antibodies contribute to seizure generation and the exact effect of LGI1 antibodies on AE remains largely unexplored. In recent research, LGI1 antibodies were found to be pathogenic in a passive transfer mouse model where patient- or control-derived IgG was transferred into the cerebral ventricle (9). Mice infused with LGI1-IgG exhibited memory impairment which partially reversed after stopping the infusion. Notably, no spontaneous seizures were observed in this model (9) However, LGI1-IgG did lead to significant reductions in the density of both total and synaptic Kv1.1 potassium channels and α-amino-3-hydroxy-5-methyl-4-isoxazolepropionic acid receptor (AMPAR) clusters resulted from the interference of LGI1 interactions with presynaptic ADAM23 and postsynaptic ADAM22 proteins. This disruption also led to increased presynaptic excitability and glutamatergic transmission, as evidenced by *in-vitro* experiments (10) but not sufficient to cause spontaneous seizures *in-vivo*. Further investigations are needed to delve into this aspect and its relevance to the clinical phenotype of LGI1- antibody associated disorders.

In this study, we attempted to determine the role of human anti-LGI1 auto-Abs in epileptogenesis by developing a translational rodent model for LGI-1-IgG that would allow for studying pathophysiology with clinical relevance and to compare it to the pathogenic consequences of anti-NMDAR-auto-Abs that had earlier been shown to contribute to the epileptogenesis (5–8). To test the hypothesis that, LGI1-antibodies may lead to increased seizure susceptibility *in-vivo*, we developed an antibody-mediated AE rat model for LGI-1gG; in which either anti-LGI1 or NMDAR auto-Ab containing total IgG obtained from blood samples of AE patients were passively transferred into the rat brain. The functional impact of each purified neuronal-surface auto-Ab on the development of a validated animal model of AE was evaluated in terms of electroencephalographical (EEG) changes, seizure susceptibility following pentylenetetrazole (PTZ) administration and behavioral manifestations.

## Materials and methods

We used non-epileptic Wistar rats (n=34) that were 3–6 months old (weighed 250-350 g) from breeding colony of Aziz Sancar Institute of Experimental Medicine. Animals were maintained under standard laboratory conditions on a 12/12-h light/dark cycle, with ad libitum access to food and water. The study was approved by the Animal Ethics Committee of Istanbul University (Ethics number:1107593) conforming with the EU Directive 2010/63/EU for animal experiments. Every effort has been made to reduce the number of animals for experimentation and minimize pain and distress in animals.

### Patient characteristics and purification of IgG

Serum samples containing anti-NMDAR (n=3) and anti-LGl1 (n=3) auto-Abs were obtained from blood samples of patients with autoimmune encephalitis. Three healthy individual (age range 28-55; 1 man/2 women) sera were used as negative controls. Both NMDAR and LGI1 encephalitis patients fulfilled the clinical criteria for definite autoimmune encephalitis and NMDAR-IgG positive patients also fulfilled the criteria for definite NMDAR encephalitis (4).

NMDAR and LGI1 antibodies were detected in both serum (end-point titers 1:400-1600) and cerebrospinal fluid (CSF) samples of NMDAR (age range 25-52; 1 man/2 women) and LGI1-encephalitis (age range 32-61; 2 men/1 woman) patients, respectively. Serum and CSF samples were tested for other well-characterized AE antibodies (anti-CASPR2, -AMPAR, -GABABR, -glutamic acid decarboxylase, -Hu, -CV2, -Ma2, -Ri and -amphiphysin) and found negative. Healthy control (HC) sera did not show any well-characterized antibodies, as well. Antibody tests were conducted by commercial cell based, immunofluorescence or immunoblot assays, as required (Euroimmun, Luebeck, Germany). All autoimmune encephalitis patients had focal and/or generalized tonic clonic seizures. Other clinical features were short-term memory loss, delusions, hallucinations, autonomic symptoms, disorientation and coma. In all patients, CSF analysis showed increased protein concentration and lymphocyte counts and EEG examination showed focal or generalized slow waves. Cranial MRI was normal in 1 each patient with NMDAR and LGI1 encephalitis, whereas the remaining patients showed bilateral medial temporal lobe hyperintensity. None of the patients showed a tumor in whole-body CT imaging. Sera were obtained within the first week of the onset of clinical episode and stored at −80°C freezer until use. None of the patients or healthy controls were under any sort of treatment during sampling. Patients with coexisting neurological/systemic disorders or pregnancy were not included. Pooled IgG obtained from healthy individuals and sterile 0.9% NaCl solution (normal saline) were used as controls. Written consent was obtained from the patients to collect blood samples, and ethical approval was obtained from the Istanbul University Faculty of Medicine Clinical Research Ethics committee in order for the serum samples prepared from blood to be used for research purposes in the future (Ethical Approval Details - Project name: Investigation of autoantibodies in autoimmune subgroups of epilepsy patients. Issue: 408/2013).

Three pooled serum samples were obtained for NMDAR encephalitis, LGI1 encephalitis patients and healthy controls. IgG was isolated from pooled sera using a protein A-Sepharose CL-4B column (Sigma-Aldrich, St. Louis, Missouri, USA), as previously reported (11, 12). The IgG-containing solution was then dialyzed against PBS and filter sterilized. IgG isolation was confirmed by demonstration of IgG bands at the expected molecular weight range by gel electrophoresis. Protein concentrations of purified IgG solutions were measured by Bradford method. NMDAR- and LGI1-immunoreactivity of purified IgG was confirmed by cell-based assays (Euroimmun) (**Supplementary Figure 1**) and characteristic IgG-binding pattern of these samples was shown by indirect immunohistochemistry using frozen rat brain sections, as described previously (13, 14) (**Supplementary Figure 2**).

### Stereotaxic surgery

Rats were anesthetized with ketamine; (100 mg/kg; i.p.) plus xylazine (10 mg/kg; i.p.) and placed into a stereotaxic frame. A guide cannula (C313G, Plastic’s One Inc., Roanoke, VA), into the lateral ventricle, (AP:-0.8, ML:-1.5, V:-4.1 mm) (15) and recording electrodes over the left frontal (AP:+2, ML: ± 3.5mm) and occipital cortex (AP:-6, ML: ± 4 mm) were implanted then fixed in position by applying dental cement around. Rats were allowed a one-week recovery period before the start of the antibody infusions.

### Intracerebroventricular (ICV) infusions

**Figure 1:**
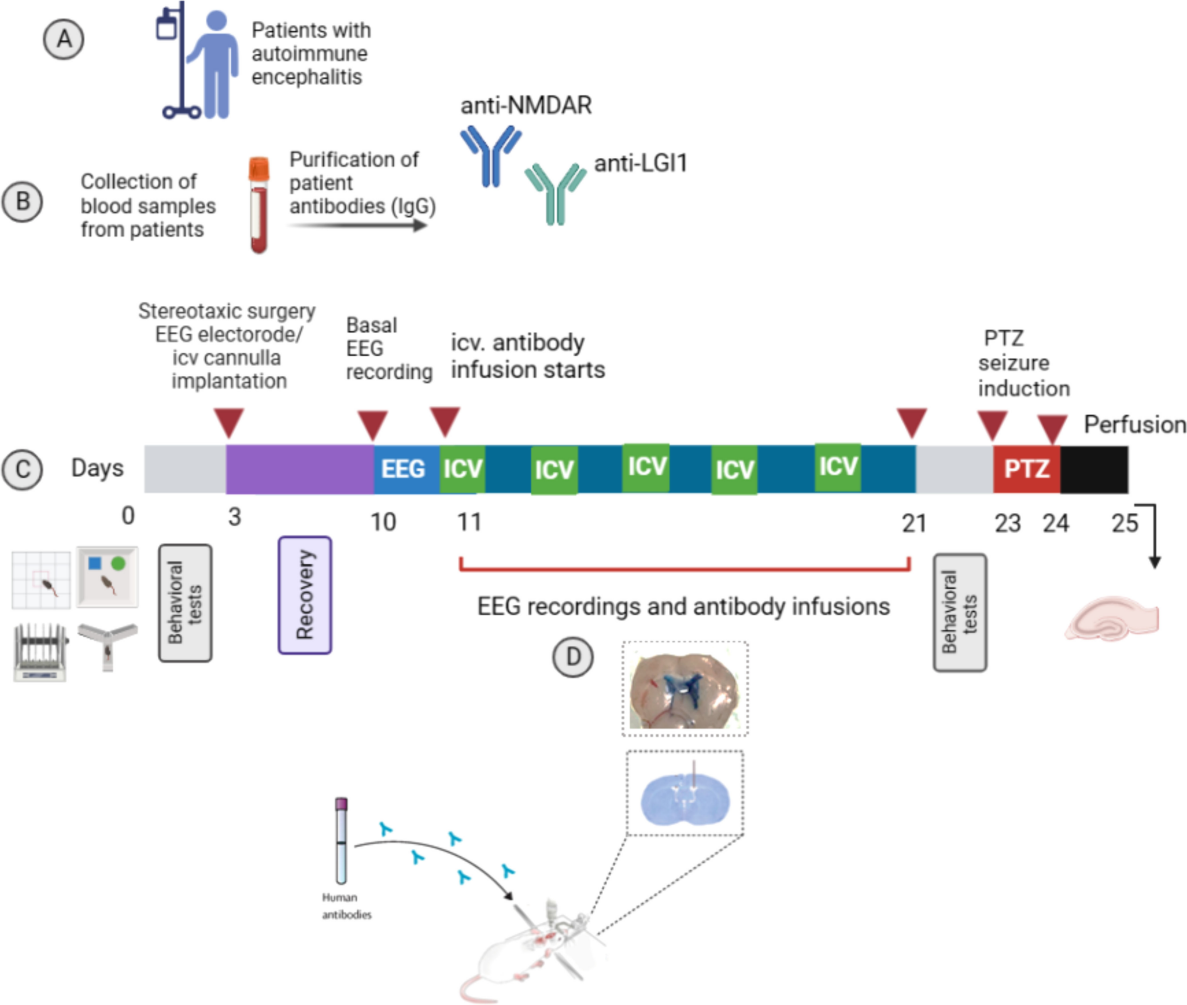
Experimental design. Experimental protocol to assess the role of NMDAR and LGI1 antibodies in in a passive transfer rat model of autoimmune encephalitis (AE). Model based on the autoantibodies obtained from blood samples of AE patients **(A)**. Total IgG obtained from the pooled sera of NMDAR and LGI1 antibody positive patients with epileptic seizures and healthy subjects was purified **(B)** and applied chronically every other day for 11 days into the cerebral lateral ventricle. Antibody infusions were performed on day 11, 13, 15 and 17 and 19 (as presented green labelled ICV). On days without infusion (10, 12, 14, 16, 18, 20th days, as presented blue). **(C)**. Spontaneous seizure development was followed by recording cortical electroencephalography (EEG) on consecutive days and after infusion administration for 2h. Behavioral tests for memory (novel object recognition test (NOR) and Y-maze) and locomotor activity (open field and rotarod tests) were applied to the animals before the antibody infusions started and at the end of the infusions. Then, pentylenetetrazole (PTZ) was administered at a convulsive dose (45 mg/kg) intraperitoneally in order to detect possible changes in seizure susceptibility. Immunohistochemistry was done for assessment of hippocampal astrocyte proliferation and expression intensity of target NMDAR and LGI1 antigens. Coronal brain section showing the appropriate ventricular placement of the guide cannullae **(D)**. Created with Biorender.com.

Animals were habituated for 20 min before infusions were performed through an internal cannula (C313I, Plastic’s One Inc., Roanoke, VA) attached to a 10-μl Hamilton syringe then EEG was recorded for 2 hours following the antibody infusions. 5 μl of purified IgG from either NMDAR antibody-positive, LGI1 antibody-positive patients or healthy controls was slowly injected over 20 min into the right cerebral ventricle via a microinfusion pump. To ensure that the antibodies were fully delivered into the ventricle, the internal cannula was kept at the injection site for at least 10 min. An equal volume of sterile saline was also delivered at a rate of 5 μl/20min to another group of rats as negative control. Infusions were continued once every two days for ten days (**Figure 1**). At the end of the tenth day, each animal was delivered with a total of 25 μl of antibody pool, which was equivalent to approximately 150-225 μg of antibodies. Appropriate ventricular placement was assessed in randomly selected rats injecting 5 μl of 1% methylene blue through the internals inserted into the guide cannula. The methylene blue traces were examined in the ventricles system to confirm the injection locations (**Figure 1D**).

### Seizure induction

To test seizure susceptibility, a subconvulsive (45 mg/kg) dose of pentylenetetrazol (PTZ; Sigma) was given intra-peritoneally (5, 16) and the rats were observed for 60 min while recording and observing PTZ-induced acute seizures. Seizures were classified according to the Racine scale (16) as follows: stage 1, behavioral arrest, chewing, and eye blinking; stage 2, stage 1 plus rhythmic head movements and head nodding; stage 3, unilateral forelimb clonus; stage 4, bilateral forelimb clonus and rearing; stage 5, falling and clonic convulsion. Seizure stage, stage 2 seizure duration, motor seizure duration and total seizure score were analyzed; the total seizure score was calculated at the end of the 10-min observation period, with a score of 2, 3, 4 or 5 given for each stage 2, 3, 4 or 5 seizure. The observation period was video recorded to allow subsequent video-EEG matching of observed events with EEG signatures.

### EEG acquisition and analysis of seizure patterns

EEG signals from the subdural screw electrodes were amplified through a BioAmp ML 136 amplifier, with band pass filter settings at 1–40 Hz, using Chart v7 program (PowerLab8S ADI Instruments, Oxfordshire, U.K). EEG was analyzed by observing the EEG trace and video recordings simultaneously to identify electrographic and behavioral seizures. Seizures were defined as rhythmic EEG activity lasting at least 5s or longer that exceeds the baseline amplitude by at least threefold.

### Behavioral assays

Behavioral tests for memory (novel object recognition test (NOR) and Y-maze) and locomotor activity (open field and rotarod tests) were applied to the animals before the antibody infusions started (on day 0) and at the end (on days 21-23) of the ICV infusions (**Figure 1**; See **Supplementary Method** for detailed description). For all variables obtained by behavioral tests, percentage change of each variable after IgG infusion was calculated using the formula (after infusion – before infusion * 100). The percentage change values were compared among treatment arms by statistical analysis.

### Immunohistochemistry

Immunohistochemical analysis was done to investigate whether LGI1 autoantibodies reduce the expression of their target antigen in a similar fashion as the NMDAR autoantibodies. Astrocytic activity was also assessed since previous studies have shown enhanced glial activity upon administration of AE samples (17, 18). At the end of the seizure induction protocol, rats were deeply anesthetized with sevoflurane and transcardially perfused with 0.1M phosphate-buffered saline (PBS). Isolated brains were post-fixed by immersion in 4% PFA for 1 h at 4°C, cryoprotected with 30% sucrose for 24 h at 4°C before sectioning at 7 μm on cryostat. First, peroxidase blocking was performed with 0.3% H_2_O_2_ to brain sections for NMDAR and GFAP assays. After washing with PBS, the sections were blocked with 10% normal goat serum (NGS) in PBS for 1 hour. Then, sections were incubated in +4°C with anti-NMDAR-NR1 subunit (PA3-102, Thermo Fisher Scientific, US; 1:250), anti-LGI1 (ab30868, Abcam, US; 1:200); GFAP (GTX40996, GeneTex, US; 1:200) primary antibodies diluted in 5% NGS in PBS. Immunoreactivity of the commercial LGI1 antibody was tested by cell-based and immunofluorescence assays (**Supplementary Figures 2, 3**). After incubation, the sections were washed with PBS and incubated at room temperature with HRP-conjugated secondary antibodies for NMDAR and GFAP (ab6721, Abcam; 1:1000 and ab6789, Abcam; 1:500, respectively) and Alexa Fluor 488-conjugated secondary antibody for LGI1 (ab150077, Abcam, 1:500) assays diluted again in 5% NGS in PBS. After PBS washing, diaminobenzidine was added to the tissues to examine the immunoreactivity under microscope for NMDAR and GFAP assays. Counter-staining was done with hematoxylin (NMDAR and GFAP) or DAPI (4’,6-diamidino-2-phenylindole) (LGI1). The images were processed using Image J (National Institutes of Health, Bethesda, MD). To ensure the diffusion of human antibodies in the hippocampus, frozen rat brain sections were incubated with biotinylated anti-human-IgG antibodies (Vector Laboratories, Newark, CA, USA), followed by the avidin-peroxidase and diaminobenzidine (19–21) (**Supplementary Figure 4**).

### Statistical analysis

Statistical analysis was performed using Graphpad Prism 9.3 (GraphPad, La Jolla, CA). All quantitative data in the text and figures are expressed as mean ± SEM. The statistical tests were chosen alternatively depending on parametric distribution. Groups were compared by Mann-Whitney U or one-way analysis of variance (ANOVA) tests, as required. The percentage changes of behavioral parameters were evaluated between groups by one-way ANOVA or Kruskal-Wallis test. Tukey’s or Dunn’s multiple comparison test was used to compare the inter-group differences. For correlation analysis Pearson or Spearman correlation tests were used, as required. *p* values of < 0.05 were considered statistically significant.

## Results

To determine whether patient-derived LGI1-IgG have a pathogenic role in epileptogenesis, we developed a passive transfer rat model in which we infused either pooled anti-LGI1 positive IgG (LGI1-IgG group, n=9) or pooled anti-NMDAR positive IgG (NMDAR-IgG group, n=8) containing total IgG obtained from blood samples of AE patients into the rat brain (**Figure 1**). We considered NMDAR group as positive control groups since the pathogenic effects are previously established (5, 8). Animals receiving total IgG collected form healthy individuals (HC-IgG, n=10) and saline (n=7) were considered as negative control groups. The animal model protocol is described in Methods and **Figure 1**. Briefly, total IgG obtained from the pooled sera of NMDAR and LGI1-IgG positive patients with epileptic seizures and healthy subjects was applied chronically every other day for 11 days into the cerebral lateral ventricle. Spontaneous seizure development was followed by electroencephalography. Behavioral tests for memory and locomotor activity were applied before and after the antibody infusions. PTZ was administered intraperitoneally to evaluate seizure susceptibility. Immunohistochemistry was performed for assessment of hippocampal astrocyte proliferation and expression intensity of target NMDAR and LGI1 antigens.

### Seizure susceptibility increased upon administration of purified IgG in both LGI1-IgG and NMDAR-IgG groups

**Figure 2:**
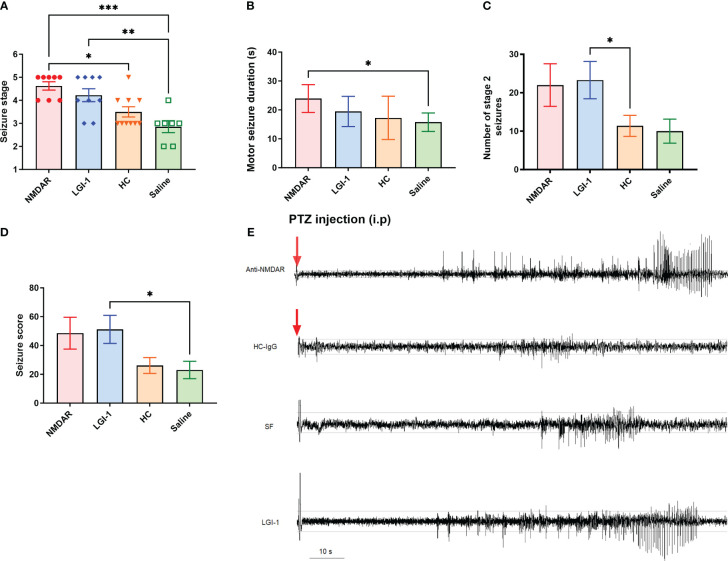
Administration of pooled IgG obtained from NMDAR and LGI1 encephalitis patients increased seizure susceptibility. Following i.p. injection of PTZ, seizure stage **(A)** was significantly higher in the NMDAR (n=8) and LGI1 (n=9) groups compared to healthy control- IgG (HC) (n=10) and saline infused control groups (n=7). Motor seizure duration **(B)** induced by acute PTZ administration was higher in NMDAR group compared to the saline infused control group. The total number of stage 2 seizures **(C)** were higher in auto-Ab groups compared to control groups. A higher total seizure score **(D)** was seen in the LGI1 group compared to saline group. **(E)** Representative EEG of ictal events recorded from a cortical electrode in animals receiving anti-NMDAR containing IgG, HC-IgG, saline and anti-LGI1 containing IgG. PTZ induced convulsive stage 4 and 5 seizures with a higher number of brief stage 2 seizures consisting of spike-and-wave activity on the EEG that precedes convulsive seizures in NMDAR and LGI1 Auto-Abs infused groups. Red arrow shows the PTZ injection time point. Horizontal guide lines show the 0.1 mV and -0.1 mV amplitude values of the EEG signal. Data were expressed as mean ± SEM. *p<0.05; **p<0.01; ***p<0.001.

No spontaneous seizure activity was recorded during the antibody infusions as well as the 10 days of observation period of non-epileptic Wistar rats treated with anti-NMDAR and anti-LGI1 IgG containing total IgG, as reported in previous studies (5). 45 mg/kg PTZ injection produced seizures (stage 2 to stage 5) in all animals which were manually scored by a blinded observer. Seizure stage was significantly higher in the LGI1-IgG and NMDAR-IgG groups compared to HC-IgG and saline infused control groups (F (2, 22) = 9.57 p<0.0001; **Figure 2A**). One-way ANOVA followed by Tukey’s multiple comparison test revealed that more severe stage 4 and 5 seizures were observed in the both antibody infused groups equally (mean seizure stage was 4.2 ± 0.27 for LGI1-IgG group; n=9 and 4.6 ± 0.18 for NMDAR-IgG group; n=8) compared to the HC (3.5 ± 0.22; n=10) and saline (2.8± 0.26; n=7) groups in which stage 3 seizures per animal was higher (**Figure 2A**). Motor seizure duration was higher in the NMDAR-IgG group (23.96 ± 1.8 s; n=8; Mann Whitney U p = 0.017) compared to the saline group (15.76± 1.4 s; **Figure 2B**). The total number of stage 2 seizures were higher in LGI1-IgG and NMDAR-IgG groups compared to negative control groups. This was significantly different in LGI1-IgG group (23.29 ± 4.8, Mann-Whitney U p=0.04) compared to HC-IgG group (11.38 ± 2.7; **Figure 2C**). Finally, the average seizure score calculated for the 10-min observation period following PTZ was higher in the LGI1 group (51.14 ± 9.7 Mann Whitney Figure p= 0.03) compared to saline group (versus 23.0 ± 6.0; **Figure 2D**). As shown in the traces sub-convulsive dose of PTZ induced more severe (stage 4 and 5) seizures with a higher number of brief stage 2 seizures consisting of spike-and-wave activity on the EEG that precedes convulsive seizures in NMDAR-IgG and LGI1-IgG groups (**Figure 2E**). This pattern was not observed in control animals those having mild stage 3 and 4 seizures.

### Memory deficits were observed in animals treated with NMDAR and LGI1 auto-antibodies

**Figure 3:**
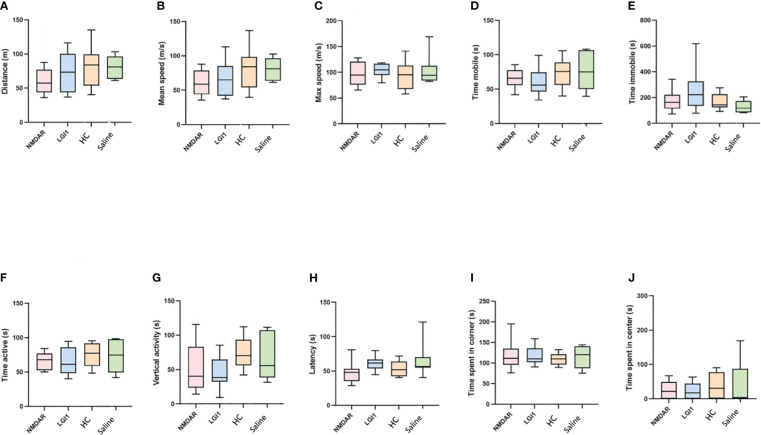
Motor functions did not change in the open field test following auto-antibody administration. In the open field test performed to measure motor functions, no significant difference was found between the groups in the parameters of distance **(A)**, mean speed **(B)**, max speed **(C)**, time mobile **(D)**, time immobile **(E)**, time active **(F)**, vertical activity **(G)** and latency **(H)** in rotarod test. In addition, no difference was observed in the open field test in terms of anxiety parameters, time spent in the corner **(I)** and time spent in center **(J)**. Data were expressed as mean ± SEM.

**Figure 4:**
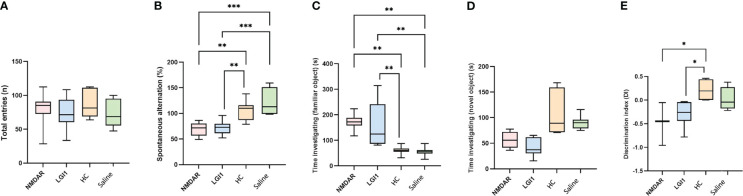
Memory deficits were observed in animals treated with NMDAR and LGI1 auto-antibodies. In the Y-maze test, total entries **(A)** was not different between groups. However, the percentage of spontaneous alternation **(B)** in the Y-maze test, which measures spatial memory, was statistically significantly lower in the NMDAR and LGI1 groups compared to the healthy control (HC) groups. In the open field test, time spent investigating familiar object **(C)** was lower however time spent investigating novel object **(D)** were significantly higher in NMDAR and LGI1 groups. Discrimination index **(E)** indicating good memory performance, was found to be lower in NMDAR and LGI1 groups. Data were expressed as mean ± SEM. *p<0.05; **p<0.01; ***p<0.001.

In the open field test performed to measure motor functions, no significant difference was found between the groups in the parameters of distance, mean speed, max speed, time mobile, time immobile, time active and vertical activity (p>0.05) (**Figures 3A–G**). Similarly, there was no difference in latency between groups in rotarod (**Figure 3H**) and total entries in Y-maze (**Figure 4A**) suggesting that the motor performance between groups did not differ (p>0.05). In addition, no difference was observed in the open field test in terms of anxiety parameters, time spent in the corner and time spent in center (p>0.05) (**Figures 3I, J**). However, the percentage of spontaneous alternation (**Figure 4B**) in the Y-maze test, which measures spatial memory, was statistically significantly lower in the NMDAR and LGI1 groups compared to the HC (p=0.0025; p=0.0063) and saline groups (p=0.0002; p=0.0005). Likewise, the time spent with the familiar object in the novel object recognition test, which measures recognition memory, was significantly higher in the NMDAR-IgG and LGI1-IgG groups than in the HC-IgG (p=0.0019; p=0.0024) and saline (p=0.0012; p=0.0015) groups, indicating poor memory (**Figure 4C**). Also, the time spent with the novel object was lower in the LGI1-IgG group than in both HC-IgG (p=0.0026) and saline groups (p=0.0264) (**Figure 4D**). Although the time spent with the novel object was found to be lower in NMDAR-IgG group compared to HC-IgG and saline groups, the significance could be only obtained for the HC-IgG group (p=0.0345). In addition, discrimination index indicating good memory performance, was found to be lower in NMDAR-IgG and LGI1-IgG groups than in the HC-IgG group (p=0.0253; p=0.0317, respectively) (**Figure 4E**). There was no significant difference between the saline group and the NMDAR/LGI1-IgG groups in terms of discrimination index (p>0.05) (**Figure 4E**). Moreover, a negative correlation was found between the seizure stage and the percentage of spontaneous alternation in the NMDAR-IgG group (r=-0.778; p=0.0394). No other significance could be obtained in the correlation analysis.

### Antibody treatment induced astrocyte proliferation and NMDAR loss in the hippocampus

**Figure 5:**
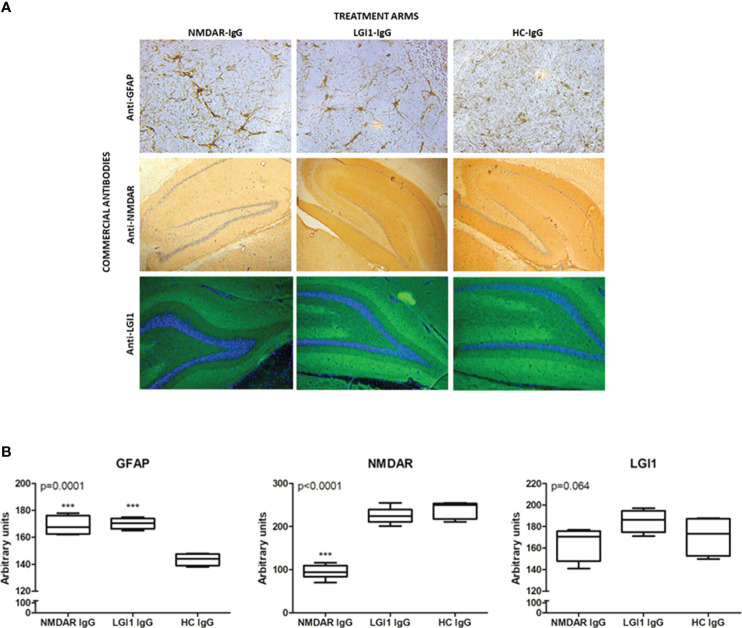
Antibody treatment induced astrocyte proliferation and NMDAR loss in the hippocampus Immunohistochemical staining of frozen sections **(A)** of rats treated icv with NMDAR-IgG (left column), LGI1-IgG (middle column) containing total serum IgG and healthy control (HC)-IgG (right column) using commercial antibodies directed against glial fibrillary acidic protein (anti-GFAP, upper row, brown color), anti-NMDAR (middle row, brown color) and anti-LGI1 (lower row, green color). Counter-staining was done with hematoxylin (blue color) in GFAP and NMDAR assays and DAPI (blue color) in LGI1 assays. Original magnification is 20x for upper row and 4x for middle and lower rows. Quantification of immunohistochemical stainings by Image J analysis **(B)**: GFAP, NMDAR and LGI1. Data were expressed as mean ± SEM p value denoted on the upper left corner of the panel is obtained by ANOVA; ***p<0.001 by Tukey’s *post-hoc* test.

NMDAR- and LGI1-IgG-groups displayed robust human IgG binding on hippocampal sections as opposed to saline injected rat brains, which did not show appreciable human IgG staining (**Supplementary Figure 4**). Sections of hippocampus were stained for GFAP immunoreactivity and assessed for typical signs of astrocyte proliferation by morphological appearance (i.e. presence of thick tortuous processes and more intense staining) (**Figure 5A**). Such staining was observed only in hippocampus of rats treated with NMDAR- and LGI1 auto-Abs (**Figure 5A**, upper row). The intensity of hippocampal GFAP staining (as assessed by Image J software) was significantly higher in NMDAR- and LGI1 groups as compared to HC-IgG group (**Figure 5B**). The intensity of commercial anti-NMDAR antibody staining in the hippocampus was significantly decreased in rats injected with NMDAR-IgG, as compared to those injected with LGI1- IgG and HC-IgG, as expected (**Figure 5A**, middle row; **Figure 5B**). The intensity of commercial LGI1 antibody staining in the hippocampus was not significantly different among different treatment arms. However, LGI1-IgG group showed trends towards exhibiting increased hippocampal LGI1 expression than NMDAR-IgG and HC-IgG groups (**Figure 5A**, lower row; **Figure 5B**).

## Discussion

In this study, we showed that passive transfer of purified IgG solutions containing NMDAR and LGI1 antibodies by ventricular infusion causes seizure susceptibility as well as memory deficits in rats. Our immunohistochemistry studies suggest that these clinical effects are likely mediated by reduced hippocampal NMDAR expression and enhanced astrocyte proliferation in the NMDAR-IgG group. Although astrocyte proliferation is also observed in the LGI1-IgG group exact molecular impact of LGI1 antibodies on the hippocampal synapses remain to be elucidated.

Several seizure-associated parameters depicted in **Figure 2** were comparable among HC-IgG and saline groups suggesting that these findings represent the background impact of the sub-convulsive dose of PTZ. Seizure severity was significantly higher in the NMDAR-IgG and LGI1-IgG groups as compared to HC-IgG and saline groups only after PTZ treatment. Our findings are congruent with several animal model studies, which have shown that single or chronic injection of NMDAR-IgG and LGI1-IgG obtained from sera or CSF of AE patients does not cause seizures in the absence of an additional seizure precipitating agent (5, 23). Therefore, at least in our rat model, NMDAR-IgG and LGI1-IgG appear to reduce the threshold for seizure induction rather than directly influencing the occurrence of seizures. In other animal model studies, infusion of mice with patient-derived anti-NMDAR monoclonal antibodies (mAb), which exhibit very high epitope binding specificity, has caused seizures (but no behavioral symptoms) without PTZ administration (17, 18). Seizures have also been induced in mice treated with NMDAR-IgG containing CSF without PTZ (6). These reports indicate that the seizure outcome in antibody-mediated animal models is determined by epitopes recognized by IgG of individual patients, the animal species used (mouse vs rat) and probably also the method by which IgG is administered.

AE associated with both NMDAR and LGI1 antibodies is typified with very high seizure prevalence (24). The seizure-inducing capacity of NMDAR- and LGI1-IgG might partially explain this seizure propensity. Clinical manifestations of AE are largely attributed to the intrathecally produced antibodies. Our study has shown that serum NMDAR/LGI1 auto-Abs generated in the secondary lymphoid organs may also contribute to the clinical symptoms if they gain access to the brain. Endogenous factors such as cytokines and inflammatory mediators found in circulation may putatively play role in fine-tuning and expression of cell-surface receptors thus leading to susceptibility to seizures and cognitive impairment in AE patients. Use of purified IgG in animal model induction largely excludes these putative non-IgG factors and emphasizes serum IgG as an important contributor of CNS findings of AE.

Our results confirm previous studies which have shown that administration of NMDAR-IgG positive serum and CSF may cause memory impairment, as demonstrated by maze and NOR experiments (8). We showed spatial and recognition memory impairment in rats in the absence of a significant alteration in motor activity and anxiety, suggesting that poor performance of NMDAR-IgG-administered rats in memory tests is not a bystander effect of the dysfunction of other cognitive and behavioral networks. Motor activity has been reported to remain intact in several previous animal models established by NMDAR-IgG (6, 17). Motor hyperactivity, seizures and lethargy have been reported in a single model using active immunization of immune competent mice with conformationally-stabilized, native-like NMDA receptors (25).

An intriguing finding was the correlation between spontaneous alternation scores and seizure stage, which cannot be attributed to PTZ that was administered after behavioral tests. This finding might be explained by affliction of common neuron groups (possibly located in the hippocampus) involved in both spatial memory and seizure induction. This argument is supported by the observation that intra-hippocampal kainate injection impairs both spatial memory and induces seizures in parallel in an animal model of epilepsy (26).

It has been well established that intrathecal administration of NMDAR-IgG reduces NMDAR expression (8). Treatment of hippocampal slices with NMDAR-IgG has been shown to diminish long-term potentiation (LTP) and increase neuronal excitability through reduced excitatory but not inhibitory neurotransmission. This finding suggests that NMDAR-IgG precipitates seizures by reducing NMDAR expression on inhibitory interneuronal populations (7). LGI1-IgG could also be inducing seizures via a similar mechanism since it is known to reduce AMPAR expression (9) However, in LGI1^-/-^ mice, increased seizure activity has been linked to increased pre-synaptic glutamate release rather than altered post-synaptic AMPAR expression (27). Likewise, an ex vivo murine brain slice study has shown that LGI1-IgG treatment leads to increased glutamate release by hippocampal neurons (28). Increased glutamergic neurotransmission has also been shown in a passive transfer model of LGI1 encephalitis (9).

LGI1 encephalitis patients present quite frequently with seizures (24). Nevertheless, the precise pathogenic mechanisms of seizures remain understudied. In two former studies utilizing intraventricular or intracerebral infusions, administration of LGI1-IgG has failed to exert spontaneous motor seizures in the absence of PTZ administration (9, 23). Thus, the most novel finding of our study was induction of seizures in Wistar rats infused with anti-LGI1 bearing IgG solution and PTZ, which to the best of our knowledge has not been reported previously. Our results also corroborate previous electrophysiological experiments demonstrating neuronal hyperexcitability with increased glutamatergic transmission, higher presynaptic release probability and reduced synaptic failure rate upon minimal stimulation and severe impairment of long-term potentiation upon intracerebral administration of anti-LG11 IgG (23).

Our study also shows that both spatial and recognition memory are impaired in rats infused with anti-LGI1 containing IgG solution. This finding confirms a single study, which has shown impaired recognition memory by NOR in LGI1-IgG administered mice (9). As indicated in two previous studies, motor functions are not altered following anti-LGI1 treatment (9, 23).

Epileptic seizure is a common manifestation of both LGI1-antibody positive encephalitis and LGI1 mutations (29). Both inborn deficiency of LGI1 and downregulation of LGI1 by short-hair pin RNA (shRNA) lead to increased neuronal network hyperexcitability and impaired synaptic plasticity in rodents (27, 29). Moreover, LGI1 encephalitis patients and experimental animals with dendrotoxin-induced Kv1.1 inhibition show remarkably similar EEG features (22). However, our immunochemistry results suggest that anti-LGI1 containing IgG does not reduce hippocampal LGI1 expression and reduced LGI1 expression is not the underlying cause of seizures in our model. Patient serum-derived LGI1-IgG might facilitate seizures putatively through increased internalization of Kv1.1 potasium channels or impaired interaction between LGI1 and ADAM22/23 proteins. It is also possible that reduction of LGI1 expression in a relatively small but functionally critical number of hippocampal neurons might be causing seizures in IgG-injected mice.

Increased GFAP reactivity was the most striking pathological feature of both anti-NMDAR and anti-LGI1 administered rats, in our study. Mild glial activation and increased GFAP reactivity have been reported in passive transfer models conducted with infusion of anti-NMDAR containing IgG or patient-derived mAbs (6, 18). Given that NMDAR-IgG reduces cerebral NMDAR expression, these findings bring forward the question whether reduced astrocytic NMDAR expression might contribute to seizure induction and memory loss. As a matter of fact, astrocytic NMDAR expression has been linked to astrocyte activation in two former studies (30, 31). In our study, we show for the first time that LGI1-IgG infusion may also lead to astrocyte activation. Astrocytic glioma cells are known to express LGI1 (32). A recent combined immunofluorescence and mass spectrometry analyses has shown that LGI1 is putatively expressed by astrocytes in mouse hippocampus (33). Thus, the impact of LGI1-IgG on glial activation pends to be further studied.

PTZ is believed to act as an antagonist to GABA_A_ receptor by directly blocking ionophores (34). PTZ increases seizure susceptibility in auto-Ab-injected rats putatively via this antagonistic activity. In support of our findings, dynamic causal modeling-mediated in depth analysis of EEG recordings of anti-NMDAR positive IgG administered animals suggests that NMDAR-Abs potentiate PTZ-induced effects in cortical microcircuitry (35). Similarly, several naturally occurring inflammatory factors that may be increased in AE (e.g. pro-inflammatory cytokines and chemokines) may putatively act as GABA_A_ receptor antagonists and reduce seizure threshold. For example, TNF-α and CCL2 have been shown to reduce neuronal GABA_A_ receptor expression (36, 37). Anti-GABAergic activity may also enhance cytotoxic T cell and macrophage responses (38). Therefore, GABA_A_ receptor agonists may potentially be used in treatment of AE as seizure-preventing and anti-inflammatory agents (39).

A limitation of our study is that it does not exclude the impact of auto-Abs directed against non-NMDAR and non–LGI1 targets that may putatively exist in the patient-derived total IgG pools administered to rats. To overcome this limitation patient-derived mAbs interacting exclusively with the target epitopes of the NMDAR have already been produced and shown to induce seizures in animal models (17). A similar approach is recommended for LGI1 antibodies in future studies.

In brief, results of our passive transfer studies conducted in rats confirm the previously established pathogenic effects of anti-NMDAR-IgG and further shows for the first time that anti-LGI1-IgG positive total IgG solution is equally capable of inducing seizures with the additive assistance of PTZ. We have also shown that anti-LGI1 auto-Abs may be involved in spatial and recognition memory impairment and astrocyte proliferation. These findings provide *in vivo* pathogenic insights into neuronal dysfunction in LGI1 encephalitis. Furthermore, our results lend support for the putative ameliorative effects of agents preventing auto-Ab-LGI1 interaction and GABAergic agonists in autoimmune encephalitis.

## Data availability statement

The original contributions presented in the study are included in the article/**Supplementary Material**. Further inquiries can be directed to the corresponding authors.

## Ethics statement

The studies involving humans were approved by the Istanbul University Faculty of Medicine Clinical Research Ethics committee (Issue: 408/2013). The studies were conducted in accordance with the local legislation and institutional requirements. The participants provided their written informed consent to participate in this study.

The animal study was approved by the Animal Ethics Committee of Istanbul University (Ethics number:1107593). The study was conducted in accordance with the local legislation and institutional requirements.

## Author contributions

ŞP: Formal Analysis, Investigation, Methodology, Project administration, Resources, Writing – original draft, Data curation. HY: Data curation, Formal Analysis, Investigation, Methodology, Software, Writing – original draft. CU: Data curation, Methodology, Writing – original draft, Resources. EŞ: Data curation, Formal Analysis, Software, Visualization, Writing – original draft. CK: Funding acquisition, Project administration, Writing – review & editing. FO: Conceptualization, Supervision, Writing – review & editing. ET: Conceptualization, Project administration, Resources, Supervision, Writing – original draft, Writing – review & editing. NÇ: Conceptualization, Supervision, Writing – review & editing, Formal Analysis, Funding acquisition, Investigation, Methodology, Project administration, Resources, Writing – original draft.
