# Supplementary material for: Antibody induced seizure susceptibility and impaired cognitive performance in a passive transfer rat model of autoimmune encephalitis
Source: Front Immunol. 2023 Nov 15;14:1268986. doi: 10.3389/fimmu.2023.1268986 (PMC10684964; doi:10.3389/fimmu.2023.1268986)
Supplement: Supplementary file 1 [file DataSheet_1.docx]

**
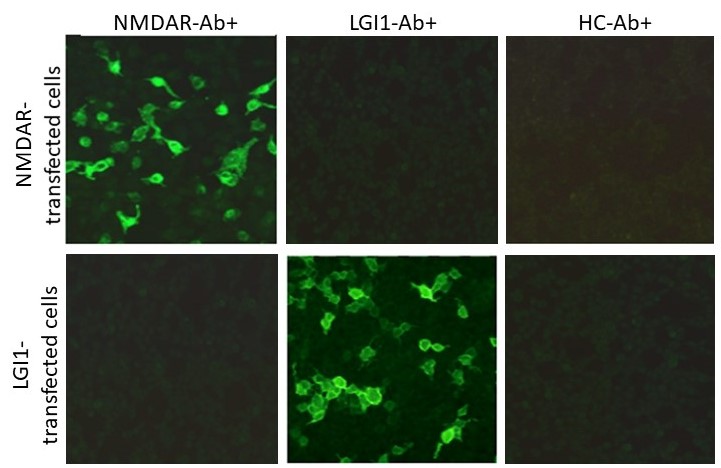
**

**Supplementary figure 1.** Reactivity (green) of IgG purified from pooled sera of NMDAR antibody (Ab) (left panels), LGI1-Ab (middle panel) positive encephalitis patients and healthy controls (HC-Ab) (right panels) with NMDAR- (upper panels) and LGI1-transfected (lower panels) human embryonic kidney (HEK293) cells expressing the NR1 subunit of NMDAR and LGI1, respectively. Note the presence of reactivity of pooled NMDAR- and LGI1-Ab positive samples with respective target antigens and absence of reactivity with pooled HC IgG. Original magnification (4x).


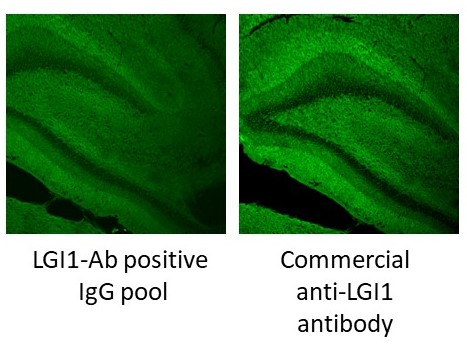


**Supplementary figure 2.** Immunolabeling of consecutive frozen rat hippocampus sections with pooled serum IgG of anti-LGI1 antibody positive patients (left) and commercial anti-LGI1 antibody (right). Original magnification (4x).

Frozen and paraformaldehyde-fixed rat brain sections were incubated with 10% goat serum for 1 h at room temperature followed by patients' sera (1:200) and commercial rabbit anti-rat LGI1 antibody (Abcam) at 1:500 dilution overnight at 4°C. Next day, sections were incubated with Alexa Fluor 488-conjugated anti-human IgG and anti-rabbit IgG (1:500 dilution for both) (Abcam) for 2 h at room temperature. Anti-human IgG-induced reactivity overlapped with anti-rabbit IgG-induced reactivity in consecutive hippocampus sections.


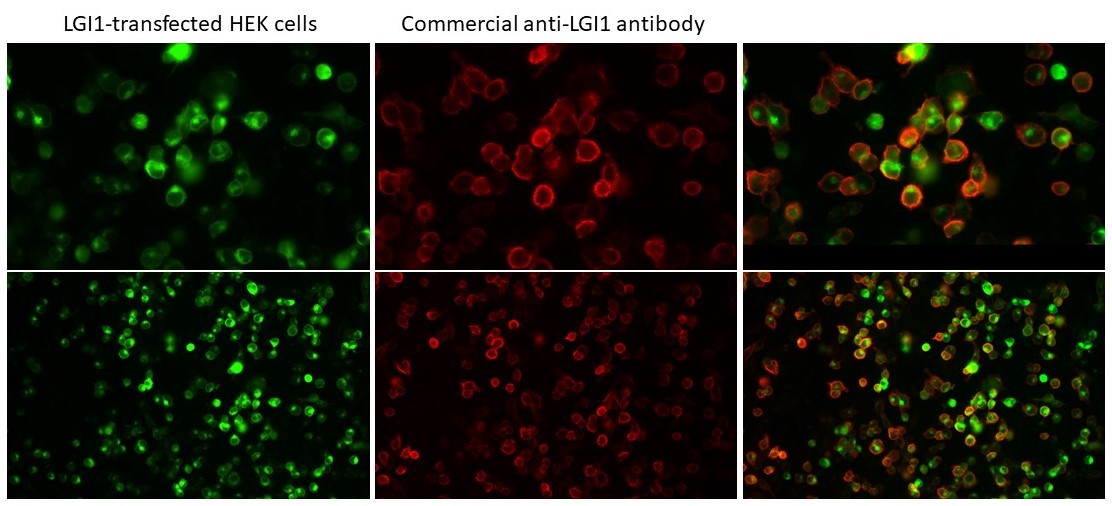


**Supplementary figure 3.** Reactivity of the commercial anti-LGI1 antibody used in immunohistochemistry analysis (and reactive with both human and rat LGI1) with transfected human embryonic kidney (HEK293) cells expressing LGI1. The reactivity of the commercial anti-LGI1 antibody (red, middle panel) co-localizes (yellow, right-panel) with HEK cells displaying the LGI1 plasmid (green, left panel). Original magnification (20x upper panels; 4x lower panels). The assay was conducted as described previously using an in-house live cell based assay (Küçükali Cİ, Şengül B, Gezen-Ak D, Dursun E, Erdağ E, Akpınar G, Kasap M, Karaaslan Z, Şirin NG, Tektürk P, Baykan B, Tüzün E. Kv5.1 antibody in epilepsy patients with unknown etiology. Epilepsy Res. 2022;182:106911. doi: 10.1016/j.eplepsyres.2022.106911).


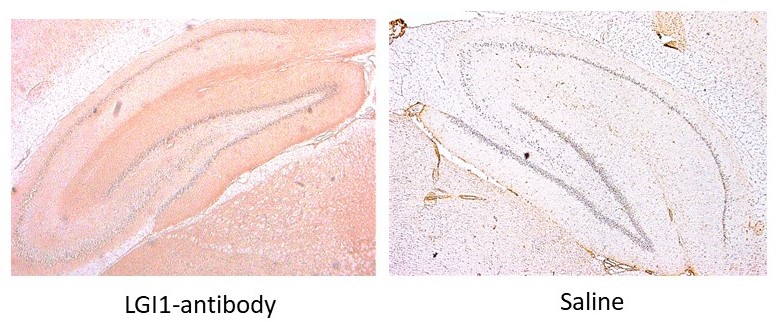


**Supplementary figure 4.** Representative staining of human IgG (brown) on sagittal sections of rat brain following intracerebroventricular administration of pooled LGI1-antibody positive IgG (left panel) or saline (right panel). Original magnification (4x). Blue, hematoxylin counterstaining. A commercial biotinylated anti-human-IgG antibody (Vector Laboratories, Newark, CA, USA) and the avidin-peroxidase method was utilized, as described previously in references 17, 18 and 21.
